# Effect of Selected Luting Agents on the Retention of CAD/CAM Zirconia Crowns Under Cyclic Environmental Pressure

**Published:** 2018-03

**Authors:** Leyla Sadighpour, Farideh Geramipanah, Akbar Fazel, Mahdi Allahdadi, Mohammad Javad Kharazifard

**Affiliations:** 1 Associate Professor, Department of Prosthodontics, School of Dentistry, Tehran University of Medical Sciences, Tehran, Iran; 2 Professor, Dental Implant Research Center, Dentistry Research Institute, Tehran University of Medical Sciences, Tehran, Iran; Department of Prosthodontics, School of Dentistry, Tehran University of Medical Sciences, Tehran, Iran; 3 Professor, Department of Prosthodontics, School of Dentistry, Tehran University of Medical Sciences, Tehran, Iran; 4 Postgraduate Student, Department of Restorative Dentistry, School of Dentistry, Shahid Beheshti University of Medical Sciences, Tehran, Iran; 5 PhD Candidate of Epidemiology, Department of Biostatistics and Epidemiology, School of Public Health, Tehran University of Medical Sciences, Tehran, Iran

**Keywords:** Zirconium Oxide, Crowns, Resin Cements, Prosthesis Retention

## Abstract

**Objectives:**

The aim of this study was to evaluate the retention strength of zirconia crowns luted with two types of resin cement under environmental pressure changes.

**Materials and Methods:**

Thirty zirconia crowns were fabricated by using computer-aided design/computer-aided manufacturing (CAD/CAM) system and were cemented by Panavia F2.0 (PAN), hand-mixed RelyX Unicem (UNH), or auto-mix RelyX Unicem Aplicap (UNA) cements on the corresponding extracted human molars. The samples were randomly divided into three groups according to the cement type. After 3000 thermal cycles, the cemented crowns were subjected to 24 pressure cycles (0 to 5 atmospheres). The retention force (N) of the specimens was measured in a universal testing machine. To normalize the retentive force, the recorded force was divided by the surface area of each tooth for measuring the retentive strength (MPa). The mean retention strengths (and forces) of the groups were compared by using one-way analysis of variance (ANOVA) and Tukey’s honest significant difference (HSD) test (α=0.05). The failure modes were also examined by using a stereomicroscope.

**Results:**

The retention values related to the evaluated resin cements were significantly different; the UNA group showed the highest retention strength (6.45±0.35 MPa) followed by the UNH (4.99±0.47 MPa) and PAN (4.45±0.39 MPa, P<0.001) groups. The adhesive failure mode was predominant in all the groups.

**Conclusions:**

The choice of resin cements and their mixing methods, which lead to differences in porosity, may affect the retention strength of zirconia crowns.

## INTRODUCTION

Modern all-ceramic materials were developed in response to the increasing demand for aesthetically pleasing and chemically and functionally durable restorations. Accordingly, zirconia-based ceramics have gained popularity due to their high strength, durability, and aesthetic appeal [1]. A clinical study has reported the 5-year survival rate of zirconia fixed partial dentures (FPDs) to be 92% [2]; however, the cumulative 5-year survival rate of a single crown was reported to be 88.8% in another study [3]. The most recurrent complications associated with zirconia-based restorations are ceramic chipping, framework fractures, and retention loss [1]. The retention of a single crown is dependent on the geometry of the preparation, fitness of the restoration, and type and quality of luting agents [4,5]. Cementation of all-ceramic restorations by adhesive resin cements is highly recommended to compensate for marginal incongruities, to promote retention, and to strengthen the restoration [6].

However, unlike silica-based ceramics, zirconia is a polycrystalline ceramic which lacks amorphous glass and is resistant to acid-etching. Consequently, no bonding is achieved by the use of conventional Bisphenol A-glycidyl methacrylate (Bis-GMA) resin cements [7]. On the other hand, in cases that involve short abutments and unusual dislodging forces, the retention must be enhanced to achieve more predictable outcomes [8,9]. The resin cements containing an adhesive functional monomer such as 10-methacryloyloxydecyl dihydrogen phosphate (10-MDP) or methacrylate phosphoric ester have been speculated to generate a durable chemical bond to zirconia-based ceramics [10]. In addition, air-abrasion by using alumina particles has been recommended to enhance the micromechanical retention [6].

Irrespective of the cement type, the cement layer is the feeblest part in adhesive-bonded ceramic restorations [11,12]; therefore, the quality of the cement layer can affect the durability of these restorations [13]. The cement thickness, flaws in the cement layer, and different mixing methods are the contributing factors that may negatively affect the bond strength [14]. The voids in the cement layer also stimulate debonding when exposed to occlusal loading, fatigue, and degradation in the oral environment [12].

Generally, luting cements consist of at least two pastes. When the pastes are mixed, air bubbles and voids can be introduced into the cement and degrade its mechanical properties [13]. Few studies have addressed the effects of mixing methods on the cement porosity and its correlation with the strength of the set cement [15,16].

The adverse effects of chemicals, moisture, pH, and physical conditions such as thermal shock and mechanical loading on the properties of dental materials and restorations have been frequently investigated in the dental literature [17]. Notwithstanding, barometric changes have been regarded as another physical condition that may influence oral tissue and dental restorations. Although the environmental pressure is approximately constant in normal life, there are situations that introduce pressure changes to the body as well as the oral cavity such as high altitude flights, diving, climbing, and working in hyperbaric conditions [18]. Although the incidence of the patients undergoing fluctuating barometric conditions is relatively low, the increasing number of people exposed to these conditions during their job or leisure time necessitates preventive methods and treatment of the side effects that those activities may introduce to the oral cavity and dentition [19]. The term barodontalgia, which appeared in the dental literature in the 1940s, refers to a pain in the orofacial region aggravated by barometric changes in the environment [20].

Dental barotrauma is the term to describe barometric-induced tooth fracture, retention loss, and restoration dislodgement [20]. With the advent of the self-contained underwater breathing apparatus (SCUBA) in the middle of the 20th century, many of the in-flight oral manifestations of barometric changes have been reported with diving as well [20]. In a survey by Jagger et al [21], it was estimated that 1% of recreational SCUBA divers may experience barotrauma. Calder and Ramsey [22] found that pressure changes could damage poorly restored teeth. Furthermore, pressure changes were shown to impair the retention of crowns cemented with zinc phosphate cements rather than the crowns cemented with a resin cement [23].

Recently, the adverse effect of the environmental pressure and luting agents on the retention of a root canal post has been investigated [24]. However, there is no evidence on how the mixing methods of resin cements may affect the retention of zirconia-based crowns during environmental pressure changes. Therefore, the purpose of the present study was to examine the retention of a computer-aided design/computer-aided manufacturing (CAD/CAM) zirconia crown luted with two types of resin cement with different mixing methods under environmental pressure variations. The null hypothesis was that the two cement types have no effect on the retention of zirconia crowns.

## MATERIALS AND METHODS

Thirty human maxillary molars, extracted within a single month due to periodontal or orthodontic treatments, were selected for the study. Informed consent was obtained from all the patients according to the protocol of the Clinical Research Ethics Board of Tehran University of Medical Sciences (no. 21409). Only the teeth without caries, cracks, or excessive wear were included in the study. The teeth were cleaned and stored in 0.1% Chloramine-T solution for two weeks and in distilled water thereafter. To ensure a consistent tooth size, the mesiodistal and buccolingual aspects of the teeth were measured by using a digital caliper (Series 500 Mitutoyo, Tokyo, Japan) with an accuracy of 0.01 mm. Only the teeth within 1 mm of the mean size were selected. One-way analysis of variance (ANOVA) was performed to confirm that no significant difference in size existed among the selected teeth. All the specimens were mounted in an acrylic block (20×20×30 mm^3^) and were prepared for full ceramic crowns. The occlusogingival height was reduced to 4 mm, and the axial surfaces were cut by using a round-end cylinder diamond bur (ISO 838.014 D+Z, Lemgo, Germany) to remove axial undercuts. Next, a round-end tapered diamond bur (ISO 856.018, D+Z, Lemgo, Germany) was used to produce a 6-degree taper with a 1-mm rounded shoulder finish line. Initially, a cylinder bur was used in order to avoid unintended preparation errors.

**Fig. 1:**
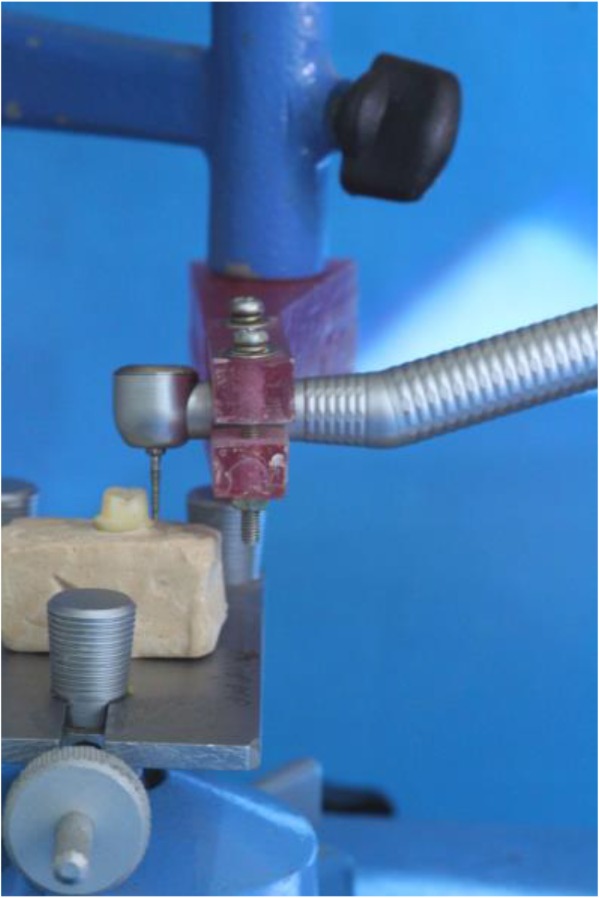
The custom-made jig mounted on the surveyor

**Fig. 2:**
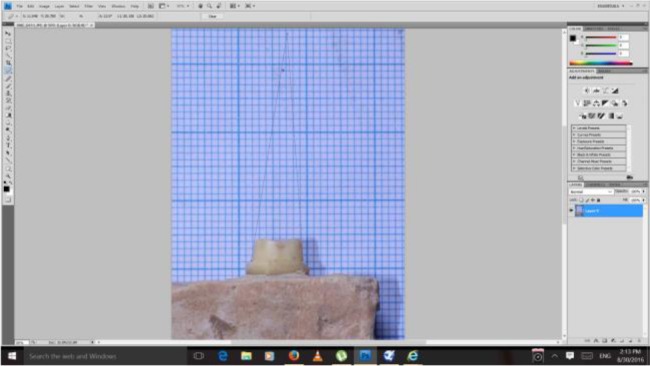
Occlusal convergence measurement by the image processing software program

**Fig. 3:**
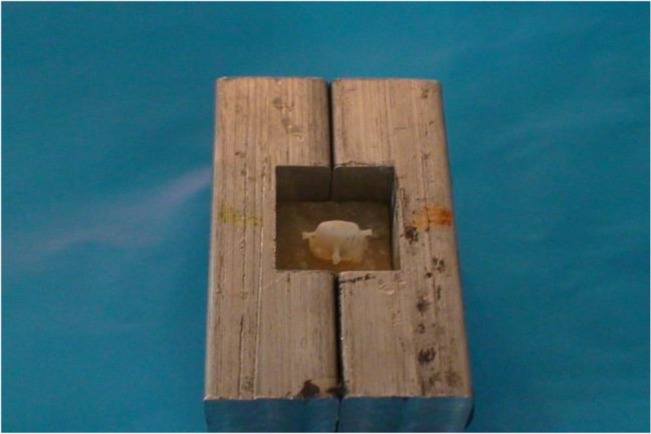
Four wings were incorporated into the coping secured in a split mold

To standardize the preparation, a custom-made device was attached to the surveyor, and a rotary instrument was used during the preparation (Fig. 1). In addition, the occlusal convergence was measured by using photographs and Adobe Photoshop CS2 image processing software (version 9.0, Adobe Systems Inc., San Jose, CA, USA) (Fig. 2). Impressions of all the specimens were made by using a polyvinyl siloxane impression material (Regular Body, Lot 95503, Elite, Zhermack, Marl, Germany) and a custom-made tray (Tray Material, Major, Moncalieri, Italy). The impressions were poured with a type IV dental stone (GC Fujirock EP, GC Corp., Tokyo, Japan). The dies on the master models were scanned by using an inLab CAD/CAM Optical Scanner (Tizian, Schütz Dental GmbH, Rosbach, Germany), and the zirconia copings were fabricated from pre-sintered blank blocks of partially stabilized zirconia. For the pull-out test, four wings were included in the coping design as described by Ernst et al [25] (Fig. 3). The internal surfaces of the copings were abraded by using 110-μm aluminum oxide particles at a distance of 10 mm and a pressure of 0.4 MPa for 20 seconds. The copings were adjusted to their corresponding teeth by using a disclosing silicone material (Fit Checker, GC Co., Alsip, IL, USA). The teeth were then randomly assigned to three groups (n=10) according to the cement type: Panavia F2.0 (PAN group; Kuraray, Osaka, Japan), hand-mixed Unicem (UNH group; RelyX Unicem, 3M ESPE AG, Seefeld, Germany), and auto-mix Unicem Aplicap (UNA group; RelyX Unicem, 3M ESPE AG, Seefeld, Germany). The cementations were performed according to the manufacturers’ instructions. To ensure a uniform seating pressure, a 5-kg weight was used to keep the crowns in place during the primary cement setting. The crowns were then light-polymerized for 40 seconds at each side at a distance of 1 mm with a light-curing unit (Coltolux LED, Coltène/Whaledent Inc., Cuyahoga Falls, OH, USA) set at 600 mW/cm^2^. The specimens were kept in 37°C water for one week, and then, were subjected to 3000 rounds of thermal cycling between 5°C and 55°C with the dwelling time of 12 seconds.

**Fig. 4:**
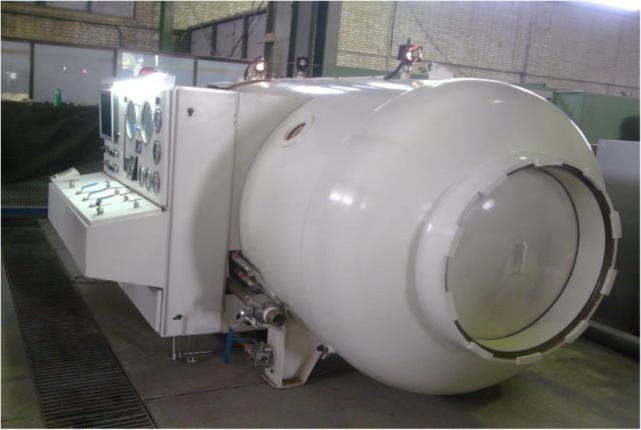
The pressure chamber

For the pressure test, the specimens were secured in a stainless steel split mold with the aid of a self-polymerizing acrylic resin (Acrosun, Betadent Co., Tehran, Iran). The pressure chamber was a custom-made device (Sadaf Recompression Chamber, Sea Industrial Organization, Isfahan, Iran) that was able to electronically control the pressure changes with an accuracy of 0.5 atmospheres (atm) (Fig. 4). The pressure cycle regimen consisted of 24 pressure cycles ranging from 0 to 5 atm at a rate of 1 atm/minute, reaching the maximum pressure of 5 atm in 5 minutes. After 5 minutes at 5 atm, the decompression phase began at a descending rate of 1 atm/minute. The test was performed according to the protocol of the Professional Association of Diving Instructors (PADI) and the U.S. Navy Diving Manual [26].

**Fig. 5:**
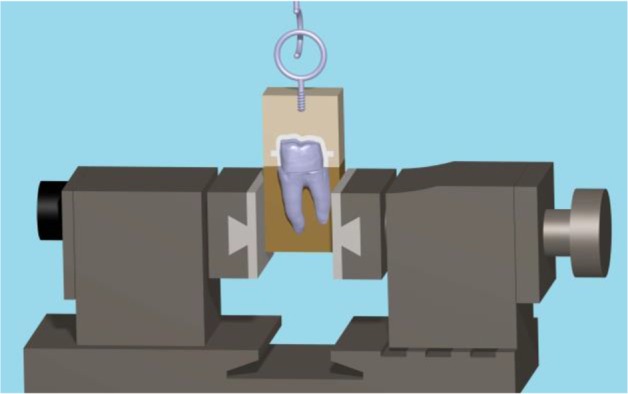
Specimen mounted in the universal testing machine for pull-out testing

To perform the pull-out test, a screw hook was implanted in an acrylic block and was glued to the crowns by using a cyanoacrylate adhesive (Superglue, RAZI Chemical Co., Tehran, Iran) to gain a uniform stress distribution (Fig. 5). The pull-out test was performed in a universal testing machine (Zwick/Roell Z050, Ulm, Germany) at a crosshead speed of 0.5 mm/minute. The retention force was sketched automatically in Newton (N) by the Zwick software program (Zwick/Roell, Ulm, Germany). In addition, the surface area of each abutment tooth was measured by adapting a tin foil and by using an engineering graph paper with 0.5-mm grids (printed from https://incompetech.com/graphpaper/gridlined/) to measure the surface area of the tin foil. The retentive strength was obtained in megapascal (MPa) through dividing the retention force by the surface area. The retentive force and retentive strength values of the test groups were analyzed for homogeneity of variances and normal distribution by the Levene’s test and Kolmogorov-Smirnov test, respectively. Afterwards, one-way ANOVA and Tukey’s honest significant difference (HSD) post hoc test were applied to examine any significant difference between the retentive force and retentive strength of the resin cements by using SPSS version 13 software program (IBM Co., Chicago, IL, USA) at the level of significance of α=0.05. The mode of failure was determined by using a stereomicroscope (Zeiss OPM1; Carl Zeiss, Oberkochen, Germany) at ×40 magnification and in four categories as follows: cement remnants predominantly on the prepared tooth (adhesive, category 1); cement remnants predominantly on the crown surface (adhesive, category 2); cement remnants observed equally over both surfaces (adhesive, category 3), and cohesive (category 4) [27].

## RESULTS

The descriptive data of the retentive force and retentive strength values are summarized in Tables 1 and 2. One-way ANOVA revealed that the cement type significantly affected the retention strength (and force) of the crowns (P<0.001). The UNA group showed the highest retention force and strength (Tables 1 and 2, P<0.001) followed by the UNH and PAN groups. The adhesive failure mode was predominant in all the groups (Table 3).

**Table 1.** The descriptive data and comparison of the mean retention force (N) among the groups

**Cement**	**Minimum** **(N)**	**Maximum** **(N)**	**Mean(SD)** **(N)**	**95% Confidence Interval (CI)**		**Sig**.^*^

				**Lower bound**	**Upper bound**	
PAN	179.10	345.90	279.19(50.14)	−248.1044	310.2756	A
UNH	279.50	424.40	325.71(46.05)	−297.1474	354.2726	B
UNA	453.70	565.30	499.89(30.58)	−489.9226	518.8573	C

PAN=Panavia F2.0, UNH= Hand-mixed Unicem, UNA=Auto-mix Unicem, SD=Standard Deviation

* Items in the columns with different letters are significantly different (CI=95%)

**Table 2.** The descriptive data and comparison of the mean retention strength (MPa) among the groups

**Cement**	**Minimum (MPa)**	**Maximum (MPa)**	**Mean(SD) (MPa)**	**95% confidence interval (CI)**		Sig.^*^

				**Lower bound**	**Upper bound**	
PAN	2.26	4.32	3.49(0.59)	−3.124	3.85	A
UNH	3.46	5.15	4.14(0.49)	−3.99	4.44	B
UNA	5.65	6.86	6.14(0.37)	−5.91	6.37	C

PAN=Panavia F2.0, UNH= Hand-mixed Unicem, UNA=Auto-mix Unicem, SD=Standard Deviation.

* Items in the columns with different letters are significantly different (CI=95%)

**Table 3.** The frequency of failure modes in the studied groups

**Cement**	**Category 1**	**Category 2**	**Category 3**	**Category 4**
PAN	4	2	4	None
UNH	4	1	4	1 (in the tooth)
UNA	3	2	3	2 (one in the crown and one in the tooth)

PAN=Panavia F2.0, UNH=Hand-mixed Unicem, UNA= Auto-mix Unicem

Category 1=Adhesive failure with the cement remaining predominantly on the crown surface

Category 2=Adhesive failure with the cement remaining predominantly on the tooth surface

Category 3=Adhesive failure with the cement remaining equally on both surfaces (tooth and crown).

Category 4=Cohesive failure

## DISCUSSION

The two resin cements evaluated in the present study significantly affected the retention strength of zirconia crowns under pressure cycles; therefore, the null hypothesis was rejected. Human teeth were used in the present study, and the coronal restorations were made to simulate the clinical situation. The primary challenge of using natural teeth is the standardization of the specimens; for this reason, the teeth with considerable differences in size were excluded from the study. All the preparations were performed with an equal height of 4 mm and a 6-degree taper with the aid of a custom-made device holding the rotary instrument and cutting bur in the same position.

In addition, the surface area of each sample was measured to calculate the retention strength [5,28]. A consistent seating pressure was provided during crown cementation by using a 5-kg weight since the seating pressure can influence the internal adaptation and the final strength of self-adhesive and self-etch cements [29]. In our study, the specimens and the test apparatus were fabricated by following the method introduced by Ernst et al [25]. In the mentioned study, a chairside tribochemical coating was applied to the outer surfaces of zirconia crowns to enhance the adhesion to the resin material inside the distracting device [25]. In our pilot experiment, the crowns were not dislodged in the pull-out device. Therefore, no further surface treatments were applied to the outer surfaces of the crowns. In the present study, the retention strength was higher in the UNA and UNH groups in comparison with the PAN group, which is not in agreement with the results of previous studies. For instance, Ernst et al [25] examined the effect of several resin cements, including Unicem and Panavia, on the retentive strength of zirconia crowns fabricated by using the Lava^TM^ system (3M ESPE AG, Seefeld, Germany). The authors found no significant difference in the bond strengths of Unicem and Panavia cements [25]. Nonsignificant differences were also found by Palacios et al [30] after evaluating the Procera® zirconia. Principally, the retentive strength of a crown could be affected by the geometry of the preparation and by the choice of luting agents. The preparation design in our study was 4 mm in height with a 6-degree taper (a 12-degree total occlusal convergence), and the crowns were fabricated by using the Cercon® zirconia system (Dentsply, DeguDent, Germany). The different materials and designs of the aforementioned studies could be the reason for the different results.

The lower bond strength of Panavia F2.0 resin cement compared to Unicem cements in our study could be explained by considering the behavior of the ED primer in the Panavia F2.0 self-etch system. The ED primer was added to the Panavia system as a co-initiator to enhance resin polymerization by providing more free radicals. However, the acidic monomer of the ED primer is incompatible with the self-curing component of the dual-curing cement, which was circumvented by adding some ionic salts to the primer, which in turn, led to the increased permeability of the primer after polymerization. Consequently, due to the increased permeability of the adhesive layer, the water from dentin or entrapped water diffused into the interfacial surface and into the adhesive layer [31]. This phenomenon, described as osmotic blistering, could impair the integrity of bonded surfaces and could compromise the quality of the cement layer [32]. The situation is exacerbated when a less-polymerized resin layer exists on top of the adhesive layer. Such a condition probably occurs when light-curing is delayed or light transmission to the resin has been compromised by light exposure through a relatively opaque restoration. As zirconia is a rather opaque ceramic, it may interfere with light transmission to the cement layer, lowering the rate of polymerization and allowing for water infusion to the interfacial surface [4]. On the other hand, Unicem cements require no dentinal pretreatment, i.e. no acidic primer is used; therefore, the incompatibility issue does not exist [4]. Furthermore, the application of pressure cycles could affect the bonded interface. In a similar study conducted by the authors [14] under the same settings, it has been shown that under the ambient pressure, the crown retention values were significantly higher in comparison with the groups in the present study. This could imply that pressure cycles may have a negative effect on the retention of crowns. The precise mechanism by which atmospheric changes affect the bonded surfaces is unknown. However, variations in the environmental pressure could affect the gas volume in body cavities according to the Boyle’s law. This law defines the correlation of the volume and pressure of a gas at a given temperature [17]. A changing pressure would inversely influence the volume of a gas. For instance, air-filled porosities in the cement layer tend to expand when the atmospheric pressure drops in circumstances like flight ascending or resurfacing after diving [17]. Consequently, cracks could develop and propagate within the cement layer, which may weaken and even disintegrate the cement layer, leading to microleakage and loss of retention [33]. Conversely, increasing the outside pressure, as in diving, could compress the porosities and air inclusions of the cement layer, which in turn, may affect the mechanical properties of the cement [18].

Since the quality of the bonded surface in the PAN group is already compromised due to water transudation within the interfacial surfaces, the extra stress of pressure changes could impose more detrimental effects on the bonded surface and could reduce the retentive strength in this group. Geramipanah et al [24] applied similar pressure cycles and found that pressure cycles decreased the retentive strength of the fiber posts luted with Panavia F2.0 cement in comparison with the posts cemented with Unicem cements. They argued that the auto-mix Unicem Aplicap could result in fewer air porosities and an enhanced integrity compared to the manually mixed version of Unicem. Although consistent results were found when the effect of mixing methods was investigated under the ambient pressure [14], this speculation should be examined by a microscopic investigation to explore the quality of the cement layer and the bonded surface, which could be regarded as a limitation of our study that deserves further investigation.

In the present study, the evaluation of failure modes did not lead to a clear conclusion since all types of failure were observed in the tested resin cements; nonetheless, the cohesive failure mode was rare. Shahin and Kern [27] stated that when cement remnants are seen on both dentin and interior surfaces of the crown (category 3), this could be regarded as a cohesive failure in the cement indicating a high bond strength since it is assumed that the bond strength to the crown and dentin is higher than the tensile strength of the cement. However, such correlation was not found in our study. This difference may be explained by the difference in the methods and materials of the two studies. In addition, restorations are subjected to thermal shocks and functional and parafunctional loadings in the oral cavity. The adverse effects of these factors have been previously investigated, though these effects were not considered in our study and could limit the generalization of the findings of the present study to real clinical situations.

## CONCLUSION

Within the limitations of the present study, the type of resin cements and their mixing methods, which lead to differences in porosity, may affect the retention strength of zirconia crowns.
